# Characterizing Tumor Response to Chemotherapy at Various Length Scales Using Temporal Diffusion Spectroscopy

**DOI:** 10.1371/journal.pone.0041714

**Published:** 2012-07-24

**Authors:** Junzhong Xu, Ke Li, R. Adam Smith, John C. Waterton, Ping Zhao, Heidi Chen, Mark D. Does, H. Charles Manning, John C. Gore

**Affiliations:** 1 Institute of Imaging Science, Vanderbilt University, Nashville, Tennessee, United States of America; 2 Department of Radiology and Radiological Sciences, Vanderbilt University, Nashville, Tennessee, United States of America; 3 AstraZeneca, Alderley Park, Cheshire, United Kingdom; 4 Department of Biostatistics, Vanderbilt University, Nashville, Tennessee, United States of America; 5 Department of Biomedical Engineering, Vanderbilt University, Nashville, Tennessee, United States of America; 6 Department of Molecular Physiology and Biophysics, Vanderbilt University, Nashville, Tennessee, United States of America; University College London, United Kingdom

## Abstract

Measurements of apparent diffusion coefficient (ADC) using magnetic resonance imaging (MRI) have been suggested as potential imaging biomarkers for monitoring tumor response to treatment. However, conventional pulsed-gradient spin echo (PGSE) methods incorporate relatively long diffusion times, and are usually sensitive to changes in cell density and necrosis. Diffusion temporal spectroscopy using the oscillating gradient spin echo (OGSE) sequence is capable of probing short length scales, and may detect significant intracellular microstructural changes independent of gross cell density changes following anti-cancer treatment. To test this hypothesis, SW620 xenografts were treated by barasertib (AZD1152), a selective inhibitor of Aurora B kinase which causes SW620 cancer cells to develop polyploidy and increase in size following treatment, ultimately leading to cell death through apoptosis. Following treatment, the ADC values obtained by both the PGSE and low frequency OGSE methods increased. However, the ADC values at high gradient frequency (i.e. short diffusion times) were significantly lower in treated tumors, consistent with increased intracellular restrictions/hindrances. This suggests that ADC values at long diffusion times are dominated by tumor microstructure at long length scales, and may not convey unambiguous information of subcellular space. While the diffusion temporal spectroscopy provides more comprehensive means to probe tumor microstructure at various length scales. This work is the first study to probe intracellular microstructural variations due to polyploidy following treatment using diffusion MRI in vivo. It is also the first observation of post-treatment ADC changes occurring in opposite directions at short and long diffusion times. The current study suggests that temporal diffusion spectroscopy potentially provides pharmacodynamic biomarkers of tumor early response which distinguish microstructural variations following treatment at both the subcellular and supracellular length scales.

## Introduction

Measurements of apparent diffusion coefficient (ADC) values using magnetic resonance imaging (MRI) provide a means to characterize the microstructure of biological tissues noninvasively, and have been widely adopted for both clinical and research applications including studies of ischemic stroke [1] and prolonged seizures [2]. In recent years, there has been increasing interest in the use of ADC measurements in oncology [3], [4]. In particular, numerous studies have shown the feasibility of ADC measurements for detecting tumors [5], for differentiating benign and malignant lesions [6], for monitoring tumor response to treatment [3], [4], [7] and for predicting therapeutic outcomes [8], [9]. Therefore, ADC values have been suggested as a promising imaging biomarker for characterizing tumor status [10].

Conventional ADC measurements use pulsed-gradient spin echo (PGSE) sequences [11], which usually incorporate relatively long diffusion times (e.g. >20 millisecond) because of practical hardware limitations. Such measurements reflect restrictions to water self-diffusion integrated over different length scales, including relatively long length scales, typically larger than a cell size, (e.g. >10 microns), and hence may be dominated by variations in restrictions at the cellular level, i.e. cellularity [12], [13] or cell density [14]. Following treatment, the measured ADC values in tumors usually increase as cells die and density decreases, and this correlation underlies the potential use of ADC as a sensitive indicator of tumor status and cell density.

However, such conventional measurements of ADC cannot distinguish structural variations *inside* tumor cells at much shorter length scales, e.g. changes in intracellular structures at subcellular level [15], [16]. According to Einstein’s relationship, the RMS displacement of water in time t_diff_ is 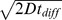. If the intrinsic diffusion water self-diffusion coefficient *D* is ∼ 3 µm^2^/ms, then for diffusion displacements to be less than a cell size, e.g. 5 microns, the diffusion time t_diff_ should be <4 milliseconds. Such short diffusion times are usually not obtainable in practice using conventional PGSE methods because short diffusion intervals demand very strong gradients. An alternative approach to obtain short diffusion times is to use oscillating gradients, in which the trapezoid-shaped gradients of the PGSE method are replaced with gradients that oscillate cosinusoidally at frequency f. Each cycle imparts a small effect of diffusion onto the MRI signal, and by varying the frequency, ADC measurements can be obtained that are sensitive to different diffusion times, and thus different length scales. When diffusion times are short (e.g. <5 milliseconds), this method provides an enhanced sensitivity to structures that hinder diffusion at subcellular length scales and thus may provide a means to detect variations in intracellular structure. When ADC values at multiple frequencies are acquired, a diffusion spectrum is obtained, which we term temporal diffusion spectroscopy [17]. Such spectra can provide novel structural information on biological tissues by reflecting restriction effects at various length scales. Temporal diffusion spectroscopy has been successfully applied to studies of tissues to demonstrate spatial heterogeneity in brain tumors [18], to probe the effects of organelle variations in cancer cells [19], to detect changes in different phases of cancer cell division cycle [20] and to monitor tumor early response to treatment [21].

In the current study, temporal diffusion spectroscopy was applied to a mouse model of colon cancer (SW620) treated with barasertib (AZD1152), a selective inhibitor of Aurora B kinase [22]. After treatment with barasertib, SW620 cancer cells develop polyploidy (more than two sets of chromosomes, i.e. contains >2 N DNA contents) and exhibit increased cell sizes, ultimately leading to cell death through apoptosis [23]. These correspond to changes in tumor structure at both subcellular and cellular levels, but the intracellular changes in particular are quite pronounced. We therefore hypothesized that the drug would increase intracellular hindrances to diffusion and thus would likely reduce ADC at short diffusion times (high temporal frequency in OGSE), in distinction from the usual pattern of ADC increase in treated tumors. If temporal diffusion spectroscopy can distinguish microstructural variations at different length scales, it can potentially be used as a new non-invasive imaging technique to monitor tumor response to treatment more comprehensively.

## Results

### Histology

**Figure 1:**
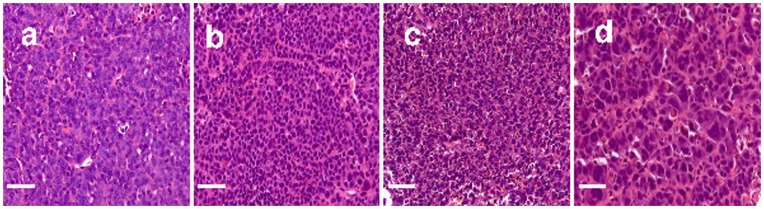
Representative histological images (40X) of tumor tissues at (a) day-0 time point without any injections; (b) day-4 in the control group (CT4); (c) day-2 in the treatment group (TX2); and (d) day-4 in the treatment group (TX4). The scale bar represents 50 microns.

Figure 1 shows representative histological images of tumor tissues at different time points. There appear no significant differences between the pre-treatment (day-0), day-4 control and day-2 post-treatment. However, there are clear increases in the nuclear sizes and cell sizes at day-4 post-treatment (see Figure 1(d)). To quantify the nuclear size changes, mean nuclear sizes of each tumor were estimated from corresponding histological images using ImageJ. The group mean nuclear sizes ± group STDs were: 7.17±0.83 µm (day-0), 7.93±0.53 µm (day-2 control), 7.21±0.48 µm (day-4 control), 9.24±1.33 µm (day-2 treatment), and 13.34±0.65 µm (day-4 treatment). Previous studies have shown that the increased nuclear size in barasertib treated SW620 cells is due to polyploidy [22]. Therefore, our histological results are consistent with previous findings and suggest that the treatment with barasertib changes molecular environment significantly, which is likely to change the diffusion properties of water molecules in the tumors at both the cellular and subcellular levels.

### ADC Maps

**Figure 2:**
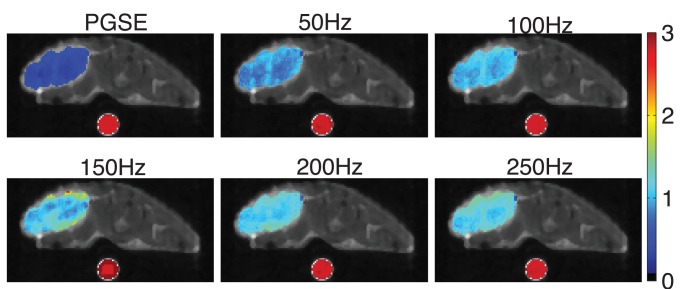
The ADC maps of a representative slice through tumor and a water phantom obtained using both the PGSE and OGSE methods overlaid on a T2-weighted MR image. The water phantom was placed beneath the mouse to monitor the accuracy and consistency of ADC measurements. Note motion artifacts of tumor and water phantom at f = 150 Hz due to a mechanical vibration.

Figure 2 shows ADC maps of representative slices obtained by the PGSE and OGSE methods at different frequencies overlaid on the corresponding T_2_-weighted image. Consistent with previous *in vivo* studies [18], [21], the ADC values obtained by OGSE methods at higher frequencies are larger than those obtained by PGSE and OGSE methods with low frequencies, which implies less restriction to water molecules at higher frequencies or shorter diffusion times [17]. A clear artifact was visible with the OGSE method at f = 150 Hz, caused by a mechanical vibration of the gradient system at that frequency (see below).

### Accuracy of ADC Measurements

**Figure 3:**
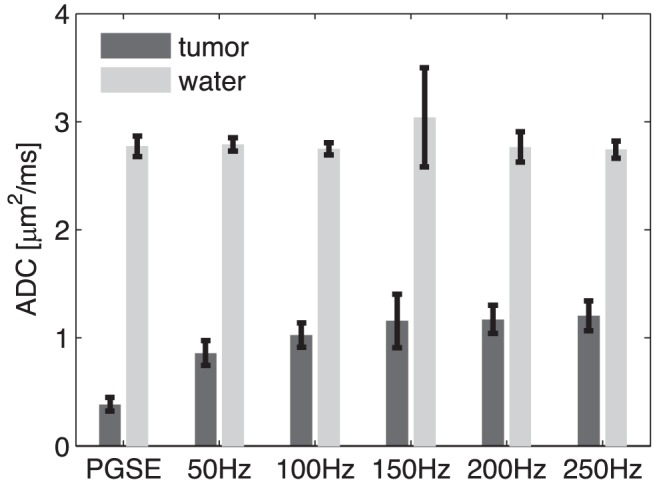
Representative ADC values of a tumor and a water phantom obtained using the OGSE method at different frequencies.

Figure 3 shows plots of the ADC values of a tumor and the water phantom from a representative mouse obtained by the OGSE method. The tumor ADC values increase with the gradient frequency as expected, showing the differential effects of larger and finer scale structures in tumors as frequency varies. The free water ADC values at different frequencies are constant, indicating that increase of ADC with frequency in tumor is not an instrument artifact. An exception is found at f = 150 Hz, in which free water ADC values show a slightly higher averaged value (∼8%) and a much larger variation. This is caused by the mechanical vibration at that frequency, which has been seen in previous experiments [24]. The motion artifacts caused by such mechanical vibration cannot be completely removed by the twin-echo 1D navigator method used in the current work [25]. A 2D navigator method may help further reduce the motion artifacts caused by such vibration and is currently under development, but note that the effect is confined to a narrow range of frequencies.

### Treatment Response Monitored by Diffusion MRI

**Figure 4:**
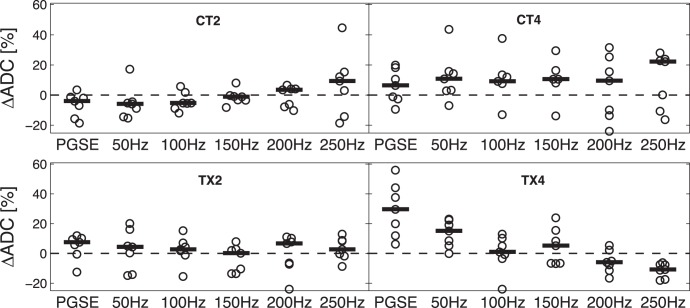
The percentage change of ADC values of all four imaging groups measured by both the PGSE and OGSE method (50–250 Hz). CT2 and CT4 are 2- and 4-day control groups; and TX2 and TX4 are 2- and 4-day treatment groups. All data points are shown as circles and solid lines indicate the mean percentage change of ADC values at each frequency in each group.

To normalize the effect of absolute ADC differences between animals, the percentage change in ADC values between pre- and post-treatment was calculated for each mouse to evaluate tumor response to treatment. Figure 4 shows the percentage change of ADC values after treatment in all four imaging groups measured by both the PGSE and OGSE method (50–250 Hz). For 2-day (CT2) and 4-day (CT4) control groups and the 2-day treatment group (TX2), the Wilcoxon signed-rank test shows p>0.05 for both the PGSE method and the OGSE method at any frequency in each of the three groups, indicating neither method detects any significant ADC change post-treatment. To further compare results obtained using different methods (the PGSE method and the OGSE method at different frequencies), the Kruskal-Wallis test was performed and provided p = 0.38, p = 0.97 and p = 0.71 for CT2, CT4 and TX2 groups, respectively, showing there is no significant difference of the detected ADC change among different methods. In contrast, the Kruskal-Wallis test provided p<0.001 in the 4-day treatment group (TX4) indicates different results were detected by different detection methods. For the PGSE method, ADC values show a remarkable increase (29% in mean with 95% CI = (13%, 46%), p = 0.02 given by the Wilcoxon signed-rank test) after treatment, indicating a reduction of diffusion restriction at cellular level. This is consistent with the histological results that SW620 tumors show a significant decrease in cell density and increase in cell size in response to barasertib treatment at 4-day post-treatment. In contrast, as shown in Figure 4, the ADC values obtained by the OGSE method at 4 days after treatment reveal important different effects from the PGSE data. At the low frequency (50 Hz), the ADC values increase (13% in mean with 95% CI = (5%, 22%), p = 0.03 given by the Wilcoxon signed-rank test) after treatment showing a similar behavior to the PGSE method. At the high frequency (250 Hz), the ADC values decrease (−11% in mean with 95% CI = (−16%, −7%), p = 0.02 given by the Wilcoxon signed-rank test) after treatment, showing a reversal of the change seen in PGSE or low frequency OGSE measurements. At intermediate frequencies (100–200 Hz), the ADC values before and after treatment did not show any significant changes. To further investigate the linear trend that the percentage ADC changes depend on gradient frequency, the Mixed model was employed. It shows p = 0.92 and 0.38 for CT4 and TX2 groups, suggesting no frequency dependence. An interesting result is that p = 0.01 for the CT2 group, which is due to one single significant ADC increase detected by the 250 Hz. However, the Mixed model provided p<0.001 for the TX4 group, indicating a significant linear dependence of the percentage ADC change on the gradient frequency (i.e. diffusion time).

### Parametric Summary of OGSE Spectra

**Figure 5:**
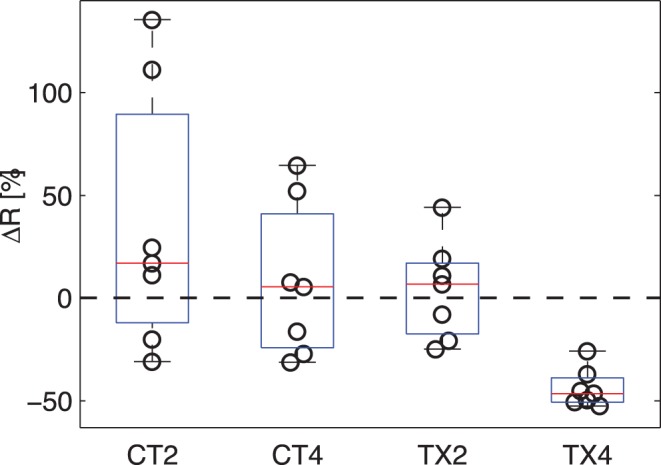
The R values in all four imaging group measured by the OGSE method. CT2 and CT4 are 2- and 4-day control groups; and TX2 and TX4 are 2- and 4-day treatment groups.

The ADC measurements from 50 Hz to 250 Hz were fit to a straight line by linear regression and the slope R was calculated. Figure 5 shows the percentage change in R values in all four imaging groups (CT2, CT4, TX2 and TX4). For both the 2-day and 4-day control groups, and the 2-day treatment group, R did not change significantly after the injections of drug/vehicle, while for the 4-day treatment group R decreased significantly after the treatment (−44% in mean with 95%CI = (−53%, −35%), p = 0.016 given by the Wilcoxon signed-rank test). The Kruskal-Wallis test gives p = 0.002 for all four groups, and the Wilcoxon rank sum test shows that the percentage change in R values of 4-day treatment group is significantly different from those of all other three groups (CT2, CT4 and TX2), indicating that ΔR was able to capture the microstructural variations of tumors in 4-day treatment group. The relatively large percentage change of R (−44% in mean) compared to ΔADC obtained by the PGSE method (%29 in mean) and OGSE at f = 250 Hz (−13% in mean) may imply that R might be a more sensitive probe for assessing tumor response because it incorporates changes in both cellularity and intracellular structures. This is consistent with previous *ex vivo* findings that the rate of ADC changes can reveal more subtle structural differences between different types of mouse brain tissues [26].

## Discussion

Previous studies have shown that SW620 tumors treated with barasertib will develop polyploidy, which accumulates a much higher fraction of cells with 4 N DNA contents (2.4-fold higher compared with controls), and 2.3-fold higher fraction of cells with >4 N DNA contents [22]. Therefore, there are massive syntheses of intracellular structures (e.g. chromosomes) in barasertib treated SW620 cells, and such microstructural variations should significantly change the molecular environment experienced by diffusing water molecules within affected cells. This treatment-induced increase of sub-cellular structures is likely to increase hindrance/restriction to water diffusion at the sub-cellular scale. In addition, the mean tumor cell size is increased and hence the cell density is decreased, which cause reductions in larger scale, cellular level restriction effects after treatment. Tumor tissue microstructure at various length scales, including both cellular and sub-cellular levels, thus changes significantly after barasertib treatment. Such treatment effects have been reported previously [22] and were confirmed by histology in the current study.

A decreased cell density can cause ADC values to increase, while increases in hindrance by intracellular structures (e.g. chromosomes and other cell organelles) can cause ADC value to decrease. These two competing factors may both contribute to the measured ADC values and the relative contribution of each depends on the diffusion time of the measurement [27]. Frequency-domain analysis of diffusion measurements shows that the conventional PGSE method with relatively long diffusion times is dominated by spectral components at very low frequencies [28]. Hence, it is more sensitive to structure at long length scales and is not able to isolate effects of intracellular structure variations (at short length scales), but detects increased ADC values post-treatment as cell density decreases. In contrast, the OGSE method provides sensitivity to various length scales with appropriate choice of gradient frequencies. When the oscillating gradient frequency is low (e.g.50 Hz in the current study), the diffusion time is relatively long and the OGSE method detects cellular level structural variations and shows a similar ADC value increase after treatment as that of the PGSE method. However, when the oscillating gradient frequency is moderately high (e.g. 250 Hz, corresponding to a diffusion time of 1 millisecond or a mean square displacement about 2 microns), the OGSE method is less affected by large scale effects and is relatively more sensitive to structural variations at the subcellular level. Therefore, after treatment, the high frequency OGSE method detects a net decreased ADC value. In the range of intermediate frequencies (100–200 Hz), the OGSE method is sensitive to intermediate length scales, and hence the effects of both decreased cell density and increased intracellular structures largely cancel each other. This is the first observation of teasing competing effects at different length scales using diffusion MRI in living tissues.

Numerous studies of cancer using the conventional PGSE method have shown that, following effective treatments, ADC values in tumors increase significantly [3], [4], [7], [12], [13], [14]. However, the current work shows that this may be an oversimplification and potentially misleading. If the intracellular restriction effects are increased, the ADC values measured at short diffusion times (e.g. 1 millisecond) will exhibit a *reversed* behavior, i.e. ADC values in tumors decrease following effective treatments. ADC values in tumors depend on the measured diffusion time and the precise length scales of pathological changes. In order to obtain more comprehensive microstructural information at various length scales within tumors, ADC measurements at different diffusion times should be used. To this end, temporal diffusion spectroscopy utilizing OGSE methods was implemented in this work to probe microstructural variations in tumors at different length levels by “tuning” gradient frequencies.

Apparent diffusion spectrum over a wide range of gradient frequencies can provide more comprehensive microstructural information about tumors, covering from very short (subcellular) to long (supracellular) length scales. However, obtaining the whole spectrum is sometimes not clinically feasible, such as the limitation of scanning time, and hence it is plausible to summarize the rich structural information contained in apparent diffusion spectra. In the current work, we proposed a new parameter, R, the rate of change in ADC values with respect to frequency. In our previous computer simulations [27], it has been shown that the cellular volume fraction can cause “the apparent temporal diffusion spectrum shifts up or down … but keeps the same shape across different frequencies”. Hence, R is suggested as a more sensitive indictor to intracellular microstructural variations than ADC values at high frequencies because the latter is affected by cellular structure as well. In the current *in vivo* study, R was found to be more sensitive to tumor response to chemotherapy, which is presumably due to increased sensitivity to intracellular microstructure variations (polyploidy). This is consistent with previous theoretical predictions [27]. It should be emphasized that over a broad frequency domain, ADC does not behave linearly with gradient frequency, so in comparing measured values of R one should be careful to use ADC measures from similar and relatively narrow frequency ranges, e.g. 50–250 Hz in the current work, for consistent fitted R results. However, due to high gradient strength demands of OGSE at increasing frequency, sampling the approximately linear regime of ADC vs gradient frequency is likely a convenient experimental approximation.

Our previous *in vitro* study shows the feasibility of temporal diffusion spectroscopy to detect intracellular structural differences during cell division cycle using a model system [20]. The current work is the first study to probe intracellular structural variations due to polyploidy following treatment using diffusion MRI *in vivo*. The results indicate that the massive macromolecules (such as chromosomes and cell organelles) increase following treatment can cause more restrictions/hindrance to diffusing water molecules inside cells, and such changes can be detected by diffusion measured at short diffusion times. We realize that these microstructural changes are caused by massive synthesis and rearrangement of macromolecules insides cancer cells, and those changes can potentially affect water diffusion in complicated ways. Thus, additional imaging and biological studies are currently underway to characterize the underlying molecular determinants of these changes and their impact on diffusion as measured by OGSE methods. Because diffusion MRI is determined by biophysical diffusion properties of microstructures, the temporal diffusion spectroscopy method is not limited to the specific tumor cell line (SW620) or specific molecular treatment mechanism (barasertib) applied in the current work. The method presented in this study may provide new insights in monitoring anticancer chemotherapies which cause massive intracellular structural variations, such as those targeting aurora kinases [29]. Moreover, polyploidy has an important role in evolution and development of cancer and other diseases [30]. The current work demonstrates the sensitivity of temporal diffusion spectroscopy to detecting polyploidy *in vivo*, and hence may potentially be used as an imaging biomarker to monitor tumor development.

The highest gradient frequency applied in the current study was 250 Hz, corresponding to a diffusion length scale <2 microns. Such frequencies can be readily achieved by conventional small animal gradient systems and more importantly, are high enough to probe variations in intracellular structure within tissues. Hence, ADC measurements at moderately high frequencies may provide a different imaging contrast from conventional PGSE and low frequency OGSE data. However, due to limitations on the available gradient strengths, the OGSE methods have been implemented at only relatively low frequencies on current human scanners [31], [32]. With the rapid development of new human gradient coils with order-of-magnitude stronger gradient strength and higher slew rate [33], OGSE methods at relatively high frequencies (e.g. 250 Hz) may be achievable in clinical studies in the near future. However, even in pre-clinical animal imaging studies alone, the OGSE method may have significant advantages over conventional PGSE methods by provide unique microstructural information inside tumor cells, which is extremely valuable to the development of novel anti-cancer treatments. Another shortcoming of OGSE methods is that a relatively long echo time is required to allow the use of longer diffusion gradient waveforms to generate sufficiently large b values, i.e. diffusion weighting. This may further reduce the signal-to-noise ratio of diffusion images, and to avoid this, relatively low b values (e.g. 400 sec/mm^2^) are suggested in measurements *in vivo*.

## Materials and Methods

### Animal and Cancer Model

All procedures were approved by our Institutional Animal Care and Usage Committee at Vanderbilt University (protocol number M/09/072). Human colon cancer cell line SW620 was obtained from American Type Culture Collection (ATCC number CCL-227). Thirty-seven female Athymic nude mice (Harlan Laboratories, Inc., Indianapolis, IN) were each injected with 1×10^7^ SW620 cancer cells into the right hind limb. Two weeks after the injection, the tumor sizes reached a defined palpable size 200–300 mm^3^ as suggested in Ref. [22]. Mice were then divided into 5 groups: 9 mice were sacrificed immediately for collection of tumor tissues; 14 mice in the 2- (TX2) and 4-day (TX4) treatment groups (N = 7 for each) received daily treatments for either 2 or 4 days respectively of 25 mg/kg barasertib dissolved in 30 µl of drug vehicle (dimethyl sulfoxide (DMSO)) per mouse; and 14 mice in the 2- (CT2) and 4-day (CT4) control groups (N = 7 for each) received drug vehicle only. The daily injection of drug or drug vehicle was administered by a single intra-peritoneal injection. Each mouse in 2-/4-day treatment/control groups was MR imaged at both pre-injection and 2-/4-day post-injection, and was sacrificed for collection of tumor tissues immediately afterwards.

### In Vivo MR Imaging

Animals were anesthetized with a 2%/98% isoflurane/oxygen mixture before and during scanning and the magnet bore temperature was kept at 32°C using a warm-air feedback system. Stretchable tapes were used to ensure the proper positioning of hind limbs and tumors and to restrain movement caused by respiration, as well as to reduce motion-induced artifacts in the image data. Respiratory signals were monitored using a small pneumatic pillow placed under the mouse abdomen and respiration gating (SA Instruments, Stony Brook, NY) was applied to further reduce motion artifacts. A doped water phantom (5 mM CuSO_4_) was placed beneath the animal with thermal equilibrium with magnet bore temperature, so the water ADC value should be constant for all measurements. Hence, ADC value of water phantom was measured to monitor the consistency of ADC measurements.

Both OGSE and PGSE sequences were implemented using a fast spin echo (FSE) acquisition on a Varian DirectDrive™ horizontal 4.7 T magnet (Varian Inc. Palo Alto, CA) equipped with a self-shielded SGRAD 115/60/S gradient system (Magnex Scientific Limited, Yarnton, Oxford, UK). A 40 mm inner diameter millipede volume coil was used for RF transmission and reception. The imaging parameters used in both PGSE and OGSE acquisitions were: diffusion gradient duration δ = 20 millisecond, separation Δ = 26.2 millisecond, echo train length = 8, echo spacing = 9.2 millisecond. Two b values were used: 0, 1000 sec/mm^2^ for the PGSE method and 0, 400 sec/mm^2^ for OGSE. Gradients with 50–250 Hz were used in the OGSE method, corresponding to diffusion times approximately from 5 to 1 millisecond. Axial slices were acquired with slice thickness 1 mm, with number of excitations (NEX) = 4. The matrix size was 128×64 with FOV = 40×20 mm yielded an isotropic in-plane resolution of 312.5 microns. Two non-phase encoded navigator echoes were acquired at the end of each echo train, and a twin-echo navigation correction was performed to reduce residual motion-induced artifacts [25].

For PGSE measurements, both positive and negative diffusion gradients were applied on alternate acquisitions, and the geometrical means of the two signals were obtained to calculate ADC value. By this means, the cross terms between diffusion gradients and the imaging/background gradients were removed [34]. The OGSE method is inherently insensitive to background gradients [35]. A potential limitation of our study is that as b = 0 images were included in the analysis of the ADC values, we cannot exclude minor contributions to ADC (or R) values from changes in perfusion. However, the gradient waveforms used in the OGSE methods are of first-order flow compensation [36]. Although there might be some small influence from higher order flows, we don’t expect perfusion effects to contribute to the OGSE signals significantly.

### Histology

Tumor tissues were collected immediately after sacrifice, fixed in 5% formalin for 24 hours and then transferred to 70% ethanol. Tissues were paraffin embedded and tumor tissue slices of 4 microns thickness were cut and then stained using haematoxylin and eosin (H&E). All histological images were obtained at a magnification of 40X using light microscopy.

A mean nuclear size of each tumor was estimated from corresponding histological images using ImageJ (Rasband, W.S., ImageJ, U.S. National Institute of Mental Health, Bethesda, Maryland, USA, http://rsb.info.nih.gov/ij/).

### Diffusion Models and Data Analysis

The PGSE method uses symmetric diffusion gradients in a spin echo sequence and records the signal changes produced by water molecule self-diffusion in the time between the gradient lobes (the diffusion time Δ). The ADC value can be calculated on a voxel-by-voxel basis using the equation
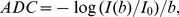
(1)where I_0_ is the T_2_-weighted signal intensity when diffusion gradient amplitude is zero, I(b) is the diffusion-weighted signal intensity with b value that can be expressed as
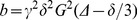
(2)where γ is the gyromagnetic ratio of proton, δ is the gradient duration, G gradient amplitude and Δ is the separation of diffusion gradients.

The OGSE method replaces the bipolar diffusion gradients in the PGSE method with oscillating diffusion gradients (usually cosine-modulated for higher sensitivity to a specific frequency and apodised to avoid infinitely-short rise time. See details in Ref. [17]). The dephasing due to diffusion occurs over each oscillating period, and the effective diffusion time is on the order of a quarter of the oscillation period and is no longer determined by the interval Δ between gradient pulses. The ADC values using OGSE methods can be calculated using Eq. [1], where the b value is given by [37]

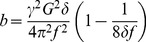
(3)where *f* is the frequency of the oscillating diffusion gradient.

Previous numerical and theoretical studies have shown that measurements of ADC at any single frequency weight the influences of different length scales differently [27]. Because restrictions exist at multiple scales, single measurements do not provide unambiguous information on any specific dimension. Ideally a complete temporal diffusion spectrum, ADC(f), provides a summary of these effects but measurements over a wide frequency range may be impractical. Instead, we introduce a new parameter
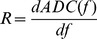
(4)which is the degree of change in ADC values when moving from low to moderately high frequencies. In the narrow gradient frequency regime, e.g. 50 Hz and 250 Hz used in the current study, this is the slope of a curve that is approximately close to linear. A similar rate of change of ADC has been suggested to reveal novel tissue contrast in white matter of fixed mouse brain [26]. We hypothesized that R might be a useful indicator of intracellular structure variations when both cellular and subcellular tumor structures are changed.

### Statistical Analysis

Regions of interest (ROIs) within tumors and water phantoms were manually selected from T_2_-weighted images. R was calculated over all frequencies using linear regression method. The Wilcoxon signed-rank test was to test if the mean percentage ADC change obtained by PGSE or OGSE at each gradient frequency was significantly different from zero, i.e. if there was significant difference between pre- and post-treatment. The Kruskal-Wallis test was applied to all percentage ADC changes of all gradient frequencies in each group to test if there was a dependence of percentage ADC change on gradient frequency. To evaluate the linear trend for the percentage change of ADC values on gradient frequencies of all groups, the Mixed model was chosen with the percentage change of ADC as the outcome, gradient frequencies as the fixed effect and animals as random effect. The random animal effect accounts for correlation between multiple measurements from the same animal (different gradient frequencies). Parameters from the Mixed model were estimated and compared. Note that Mixed model was chosen in the current work instead of a chi-square straight line fit because mixed model is more appropriate to incorporate the cluster effect due to multiple observations (at different gradient frequencies) measured from the same animal. The Mixed model analysis was performed using R2.9.2.

## References

[1] Moseley ME, Cohen Y, Mintorovitch J, Chileuitt L, Shimizu H (1990). Early detection of regional cerebral ischemia in cats: comparison of diffusion- and T2-weighted MRI and spectroscopy.. Magn Reson Med.

[2] Righini A, Pierpaoli C, Alger JR, Di Chiro G (1994). Brain parenchyma apparent diffusion coefficient alterations associated with experimental complex partial status epilepticus.. Magn Reson Imaging.

[3] Zhao M, Pipe JG, Bonnett J, Evelhoch JL (1996). Early detection of treatment response by diffusion-weighted 1H-NMR spectroscopy in a murine tumour in vivo.. Br J Cancer.

[4] Ross BD, Moffat BA, Lawrence TS, Mukherji SK, Gebarski SS (2003). Evaluation of cancer therapy using diffusion magnetic resonance imaging.. Mol Cancer Ther.

[5] Tsuruda JS, Chew WM, Moseley ME, Norman D (1991). Diffusion-weighted MR imaging of extraaxial tumors.. Magn Reson Med.

[6] Guo Y, Cai YQ, Cai ZL, Gao YG, An NY (2002). Differentiation of clinically benign and malignant breast lesions using diffusion-weighted imaging.. J Magn Reson Imaging.

[7] Chenevert TL, McKeever PE, Ross BD (1997). Monitoring early response of experimental brain tumors to therapy using diffusion magnetic resonance imaging.. Clin Cancer Res.

[8] Moffat BA, Chenevert TL, Lawrence TS, Meyer CR, Johnson TD (2005). Functional diffusion map: a noninvasive MRI biomarker for early stratification of clinical brain tumor response.. Proc Natl Acad Sci U S A.

[9] Moffat BA, Chenevert TL, Meyer CR, McKeever PE, Hall DE (2006). The functional diffusion map: an imaging biomarker for the early prediction of cancer treatment outcome.. Neoplasia.

[10] Padhani AR, Liu G, Koh DM, Chenevert TL, Thoeny HC (2009). Diffusion-weighted magnetic resonance imaging as a cancer biomarker: consensus and recommendations.. Neoplasia.

[11] Stejskal EO, Tanner JE (1965). Spin diffusion measurements - spin echoes in presence of a time-dependent field gradient.. J Chem Phys.

[12] Sugahara T, Korogi Y, Kochi M, Ikushima I, Shigematu Y (1999). Usefulness of diffusion-weighted MRI with echo-planar technique in the evaluation of cellularity in gliomas.. J Magn Reson Imaging.

[13] Gauvain KM, McKinstry RC, Mukherjee P, Perry A, Neil JJ (2001). Evaluating pediatric brain tumor cellularity with diffusion-tensor imaging.. AJR Am J Roentgenol.

[14] Lyng H, Haraldseth O, Rofstad EK (2000). Measurement of cell density and necrotic fraction in human melanoma xenografts by diffusion weighted magnetic resonance imaging.. Magn Reson Med.

[15] Xu J, Does MD, Gore JC (2009). Sensitivity of MR diffusion measurements to variations in intracellular structure: Effects of nuclear size.. Magn Reson Med.

[16] Gore JC, Xu J, Colvin DC, Yankeelov TE, Parsons EC (2010). Characterization of tissue structure at varying length scales using temporal diffusion spectroscopy.. NMR Biomed.

[17] Parsons EC, Does MD, Gore JC (2006). Temporal diffusion spectroscopy: theory and implementation in restricted systems using oscillating gradients.. Magn Reson Med.

[18] Colvin DC, Yankeelov TE, Does MD, Yue Z, Quarles C (2008). New insights into tumor microstructure using temporal diffusion spectroscopy.. Cancer Res.

[19] Colvin DC, Jourquin J, Xu J, Does MD, Estrada L (2011). Effects of intracellular organelles on the apparent diffusion coefficient of water molecules in cultured human embryonic kidney cells.. Magn Reson Med.

[20] Xu J, Xie J, Jourquin J, Colvin DC, Does MD (2011). Influence of cell cycle phase on apparent diffusion coefficient in synchronized cells detected using temporal diffusion spectroscopy.. Magn Reson Med.

[21] Colvin DC, Loveless ME, Does MD, Yue Z, Yankeelov TE (2011). Earlier detection of tumor treatment response using magnetic resonance diffusion imaging with oscillating gradients.. Magn Reson Imaging.

[22] Wilkinson RW, Odedra R, Heaton SP, Wedge SR, Keen NJ (2007). AZD1152, a selective inhibitor of Aurora B kinase, inhibits human tumor xenograft growth by inducing apoptosis.. Clin Cancer Res.

[23] Moroz MA, Kochetkov T, Cai S, Wu J, Shamis M (2011). Imaging colon cancer response following treatment with AZD1152: a preclinical analysis of [18F]fluoro-2-deoxyglucose and 3′-deoxy-3′-[18F]fluorothymidine imaging.. Clin Cancer Res.

[24] Parsons EC (2002). Nuclear magnetic resonance methods for the analysis of water diffusion in neural tissue: Yale University..

[25] Mori S, van Zijl PC (1998). A motion correction scheme by twin-echo navigation for diffusion-weighted magnetic resonance imaging with multiple RF echo acquisition.. Magn Reson Med.

[26] Aggarwal M, Jones MV, Calabresi PA, Mori S, Zhang J (2012). Probing mouse brain microstructure using oscillating gradient diffusion MRI.. Magn Reson Med.

[27] Xu J, Does MD, Gore JC (2011). Dependence of temporal diffusion spectra on microstructural properties of biological tissues.. Magn Reson Imaging.

[28] Stepisnik J (1981). Analysis of NMR self-diffusion measurements by a density-matrix calculation.. Physica B.

[29] Gautschi O, Heighway J, Mack PC, Purnell PR, Lara PN (2008). Aurora kinases as anticancer drug targets.. Clin Cancer Res.

[30] Storchova Z, Pellman D (2004). From polyploidy to aneuploidy, genome instability and cancer.. Nat Rev Mol Cell Biol.

[31] Hosey TP, Harding SG, Green HA, Ansorge RE, Carpenter TA. Diffusion Tensor Imaging, Using Oscillating Gradients to Probe Short Diffusion Times, in the Human Brain.. ; 2003 July; Toronto, Canada.

[32] McHugh DJ, Hubbard PL, Zhao S, Higgins DM, Parker GJ, et al.. Probing tissue microstructure using oscillating diffusion gradients in the human calf; 2011 May; Montreal, Canada.

[33] Aksel B, Marinelli L, Collick BD, Von Morze C, Bottomley PA (2007). Local planar gradients with order-of-magnitude strength and speed advantage.. Magn Reson Med.

[34] Neeman M, Freyer JP, Sillerud LO (1991). A simple method for obtaining cross-term-free images for diffusion anisotropy studies in NMR microimaging.. Magn Reson Med.

[35] Hong X, Thomas Dixon W (1992). Measuring diffusion in inhomogeneous systems in imaging mode using antisymmetric sensitizing gradients.. J Magn Reson.

[36] Maki JH, MacFall JR, Johnson GA (1991). The use of gradient flow compensation to separate diffusion and microcirculatory flow in MRI.. Magn Reson Med.

[37] Does MD, Parsons EC, Gore JC (2003). Oscillating gradient measurements of water diffusion in normal and globally ischemic rat brain.. Magn Reson Med.

